# Structure observation of graphene quantum dots by single-layered formation in layered confinement space†
†Electronic supplementary information (ESI) available: Detailed experimental materials, apparatus, experimental procedures and characterization data. See DOI: 10.1039/c5sc01416f



**DOI:** 10.1039/c5sc01416f

**Published:** 2015-06-04

**Authors:** Liqing Song, Jingjing Shi, Jun Lu, Chao Lu

**Affiliations:** a State Key Laboratory of Chemical Resource Engineering , Beijing University of Chemical Technology , Beijing 100029 , China . Email: luchao@mail.buct.edu.cn ; Fax: +86 10 64411957 ; Tel: +86 10 64411957

## Abstract

Graphene quantum dots (GQDs) have received much research attention due to their unique optical/electrical/magnetic features. However, the intrinsic relationship between the structure and properties of GQDs remains not fully understood, because the controlled synthesis and the accurate structure determination of GQDs are still a great challenge. Here, single-layered GQDs are successfully obtained by the hydrothermal treatment of intercalated citrate in the confined space of 2D interlayer galleries of layered double hydroxides (LDHs). The distinct molecular structures of the single-layered GQDs reveal that the blue photoluminescence of the GQDs derives from a rigid π-conjugate plane structure, which is in accordance with theoretical calculations. This work will open the door towards a quantitative understanding of the structure-property effects of GQDs.

## Abstract 2

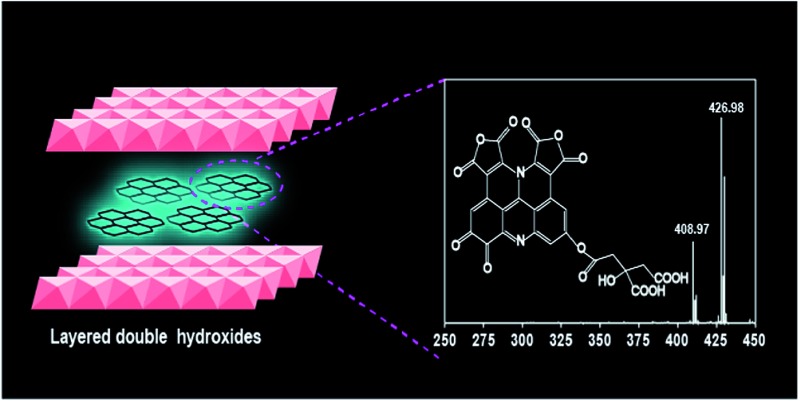
We observe the structure of single-layered graphene quantum dots prepared in the 2D confined space of layered double hydroxides.

## Introduction

Graphene quantum dots (GQDs) have made great progress, in their synthesis and in a range of potential applications in optoelectronics and the biomedical field, owing to their intriguing properties, such as strong photoluminescence, good chemical/physical robustness and excellent biocompatibility.^1^ In spite of the continuously increasing research interest in GQDs, many characteristics of GQDs, *e.g.*, the photoluminescence mechanism, have not yet been fully elucidated.^2^ Currently, there are several hypotheses on the photoluminescence origin of GQDs.^3^ For instance, Sk *et al.* suggested that the photoluminescence of GQDs was derived from the sp^2^ carbon network, and the photoluminescence property was predicted to be widely tuned by size, edge structure, shape, functional groups, defects, and heteroatom doping.^3*a*^ Pan's group proposed that the blue luminescence of GQDs might originate from free zigzag sites with a carbene-like triplet ground state described as σ^1^π^1^.^3*b*^ Lingam *et al.* attributed the photoluminescence in GQDs to the presence of edge-states.^3*c*^ Liu and co-workers explained that the blue photoluminescence of GQDs was dominated by the intrinsic state in the high-crystalline structure.^3*d*^ These ambiguous mechanisms are mainly caused by the difficulty in obtaining GQDs with an accurate chemical composition. However, current GQDs produced by two main methods (top-down and bottom-up) are mostly multi-layered with a broad distribution in both size and structure.^4^ Therefore, it is highly desirable to generate single-layered GQDs (S-GQDs),^5^ which possess an exact number of composed atoms and exact chemical structure, in order to unveil the relationship between the chemical structure and photoluminescence characteristics of GQDs.

Layered double hydroxides (LDHs) represent a major class of inorganic layered materials with tunable interlayer spaces and variable interlayer guests.^6^ The confined space of the 2D interlayer galleries of the LDH hosts leads to substantial improvements in the luminescence properties of the fluorophore ensemble.^7^ Many efforts are being devoted to prepare nanomaterials with superior physical and chemical properties by incorporating suitable precursors into LDHs through ion exchange methods or co-precipitation methods.^8^ An obvious confinement effect in the restrained interlayer environment of LDHs serves as a 2D nanoreactor, and thus the *in situ* reaction of intercalated precursors is restricted to occur in the gallery spaces. Taking into account that GQDs have molecular dimensions of several nanometers, thus LDHs should be ideal hosts to accommodate the precursors to prepare S-GQDs with a narrow size distribution.

**Fig. 1:**
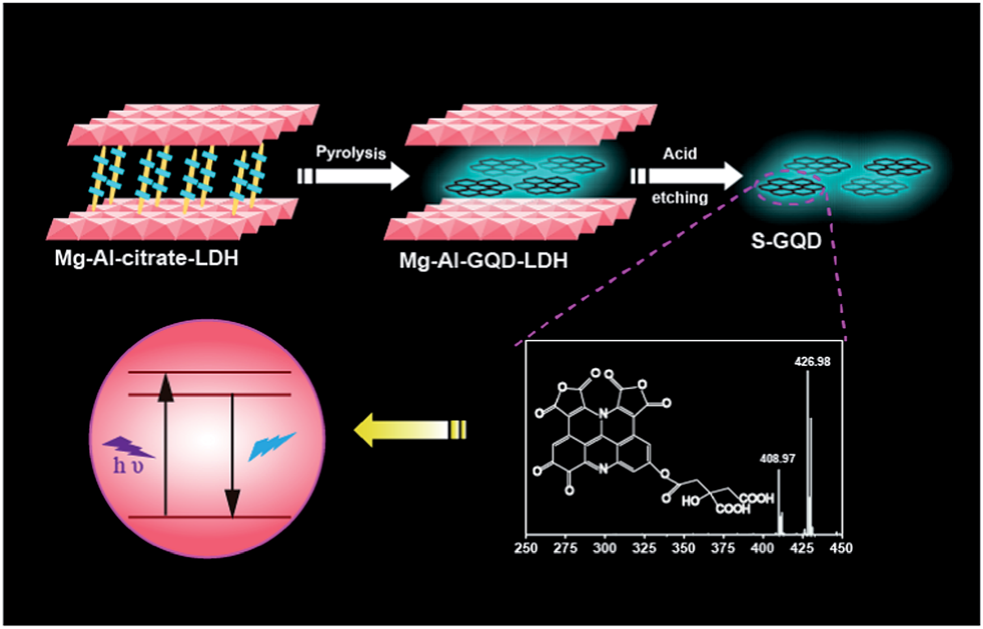
Schematic illustration of the formation of S-GQDs in the confined space of LDH.

Here we synthesized S-GQDs in a 2D confined nanoreactor of layered double hydroxides. The structure of the S-GQDs is well-maintained in the LDH interlayer with a single-layered thickness of 0.7 nm and uniform size owing to the effective limitation of *in situ* growth of the GQDs. Nuclear magnetic resonance spectroscopy (NMR) and high resolution electrospray ionization Fourier transform ion cyclotron resonance mass spectrometry (ESI-FTICR-MS) have been used to elucidate the molecular structures of the S-GQDs (Fig. 1). The distinct chemical structures of the S-GQDs provide solid evidence to support the results that the rigid π-conjugate plane of S-GQDs with a specific edge is responsible for the observed blue photoluminescence. In addition, the theoretical calculations described here gives valuable insights into the understanding of the relationship between the chemical structure and the photoluminescence characteristics of the GQDs.

## Results and discussion

### Single-layered GQDs

**Fig. 2:**
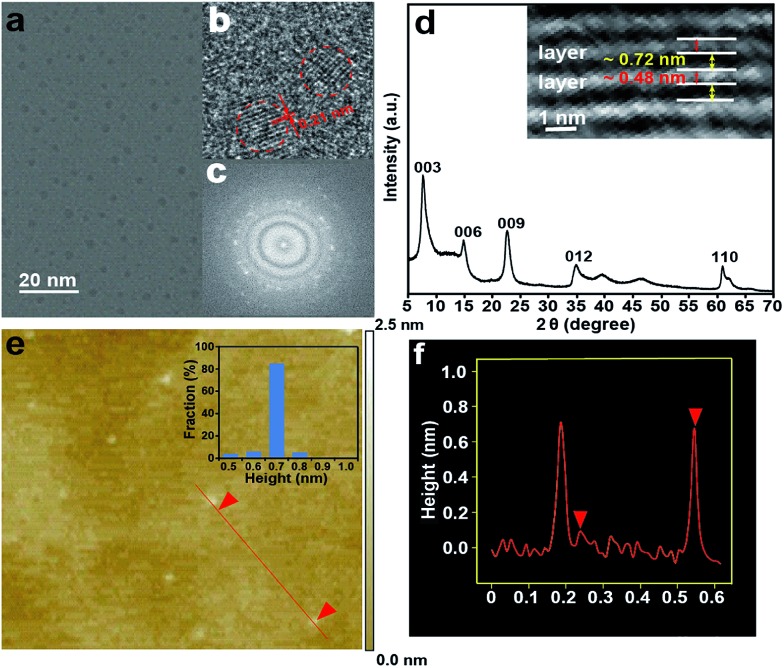
(a) TEM image of the GQDs. (b) HRTEM image of the GQDs; (c) FFT pattern of the GQDs in (b); (d) XRD pattern of the Mg–Al–GQD-LDHs. Inset is the HRTEM of the Mg–Al–GQD-LDHs; (e) AFM image of the S-GQDs on a Si substrate. Inset is the height distribution of the S-GQDs; (f) height profile along the red line in (e).

Citrate-intercalated Mg–Al-LDHs (Mg–Al–citrate-LDHs) are synthesized *via* a co-precipitation method in solutions of constant pH value. The X-ray diffraction (XRD) pattern (Fig. S1†) and Fourier transform infrared spectroscopy (FTIR) (Fig. S2†) of the Mg–Al–citrate-LDHs samples demonstrate that citrate molecules have been successfully intercalated into the LDHs with an interlayer distance of ∼0.7 nm.^9^ Next, under the optimum reaction time (Fig. S3†) and intercalated quantity of citrate (Fig. S4†), the well-defined S-GQDs are synthesized *via* the hydrothermal carbonization of organic guests of citrate in the confined space of the 2D interlayer galleries of the LDH hosts in the presence of ammonia at 180 °C for 8 h. In addition, it is found that the photoluminescence intensity of the Mg–Al–GQD-LDHs significantly increases in the presence of ammonia due to the fact that ammonia serves dual functions: an environment with high pressure for accelerating the decomposition of citrate, and as a nitrogen source (Fig. S5†).^10^ The obtained solid Mg–Al–GQD-LDHs were then etched with hydrochloric acid to give the GQD colloidal solution.^8*a*^ The transmission electron microscopy (TEM) image (Fig. 2a) shows that the as-produced S-GQDs have a narrow size distribution of 2.2 ± 0.2 nm. The lattice spacing of the GQDs imaged by high resolution TEM (HRTEM) (Fig. 2b) is *ca.* 0.21 nm, which is very close to the hexagonal pattern of graphene with d_1100_.^11^ The fast Fourier transform (FFT) pattern of the GQDs is shown in Fig. 2c. In contrast, we have also prepared GQDs from pure citrate by the same hydrothermal route, the TEM image indicates that the as-prepared GQDs have a size distribution of 3.0 ± 0.5 nm (Fig. S6a†). In addition, a Raman spectrum is employed to provide direct evidence for the graphene structure of the products.^12^ The peaks centered at 1363 and 1580 cm^–1^ are assigned to the D and G bands related to sp^2^-bonded C atoms and disordered C atoms at the edges of the S-GQDs, respectively (Fig. S7†). It is obvious that the S-GQDs have a *I*_D_/*I*_G_ ratio of *ca.* 0.7, indicating the formation of S-GQDs with high quality.^12^

The XRD pattern of the Mg–Al–GQD-LDHs is presented in Fig. 2d. The basal spacing of the Mg–Al–GQD-LDHs is *ca.* 1.2 nm, consisting of a LDH host layer with a thickness of ∼0.48 nm and an interlayer spacing of ∼0.72 nm.^13^ The HRTEM image of the Mg–Al–GQD-LDHs (the inset of Fig. 2d) presents the expected platelet morphology, with the interplanar distance of *ca.* 1.2 nm, which is in good agreement with the XRD data. On the other hand, the corresponding atomic force microscope (AFM) images of the GQDs (Fig. 2e and f) reveal that the average height of most GQDs is 0.7 nm (more than 90%), disclosing the monolayer nature of the products.^5^ However, the AFM image (Fig. S6b†) of the GQDs obtained from pure citrate reveals that the average height of the GQDs is 2.5 nm (Fig. S6c and d†), corresponding to *ca.* 4–5 graphene layers. These results prove that the size and height of the GQDs are effectively controlled inside the confined 2D galleries of LDHs, leading to the formation of high-quality S-GQDs.

### Chemical structure of single-layered GQDs

The composition and structure of the S-GQDs are characterized by different spectroscopy techniques including ^1^H and ^13^C NMR, X-ray photoelectron spectroscopy (XPS), FTIR, and ESI-FTICR-MS/MS.

**Fig. 3:**
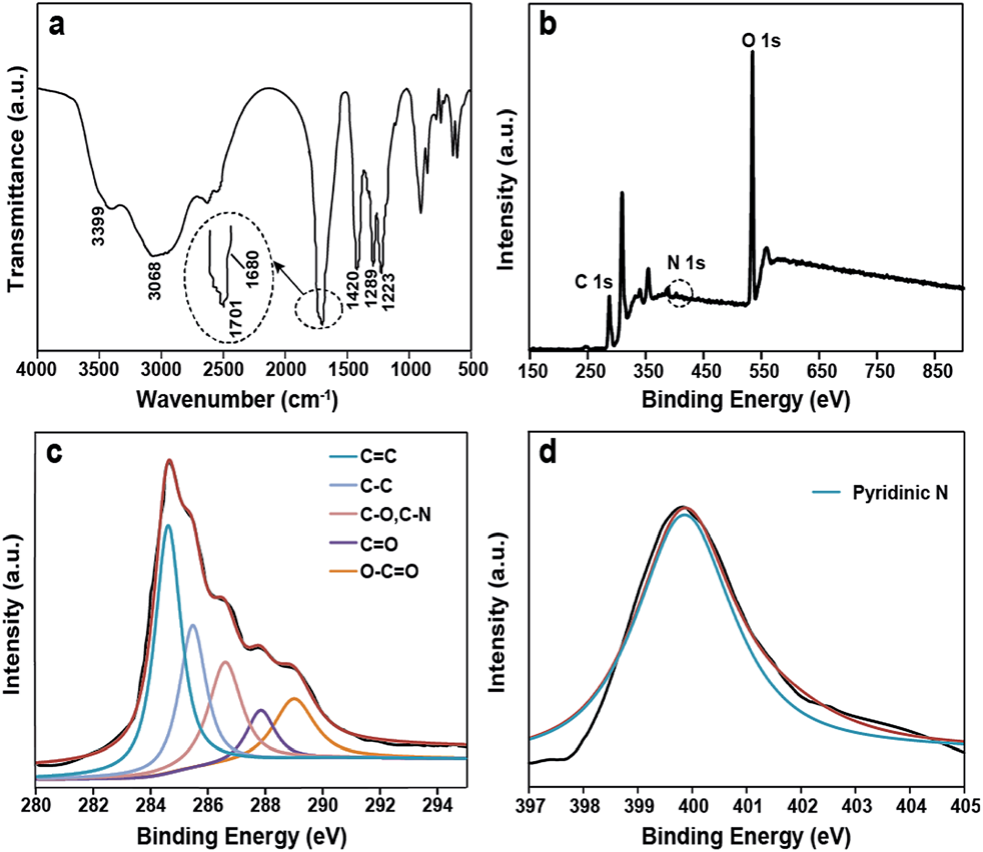
(a) FTIR spectrum of the S-GQDs; (b) full-scan XPS spectrum of the S-GQDs; (c) the C 1s XPS spectra of the S-GQDs; (d) N 1s XPS spectra of the S-GQDs.

The FTIR spectrum of the as-prepared S-GQDs shows a broad absorption band corresponding to the O–H stretching vibration (2500–3500 cm^–1^) (Fig. 3a). The absorption band centered at 1701 cm^–1^ stands for the C=O stretching vibration. The COOH stretching vibration is located at 1680 cm^–1^. The absorption bands at 1289 cm^–1^ and 1223 cm^–1^ are ascribed to the C–O–C stretching vibration. The peak at 1420 cm^–1^ denotes the existence of N-related bonds.^14^ Note that the zeta potential of the as-prepared S-GQDs is –7.17 mV, further supporting the negative-charged carboxylic and hydroxyl groups on the S-GQDs.^15^ XPS measurements are also performed to gain structural insight into the as-produced S-GQDs.^12^ Specifically, a predominant graphitic C 1s peak at 284.9 eV, an obvious N 1s peak at 399.9 eV, and an O 1s peak at 532.21 eV are recognized in the XPS spectra (Fig. 3b). In detail, the C 1s peak can be deconvoluted into five peaks (Fig. 3c), namely, 284.6 eV (C=C), 285.5 eV (C–C), 286.6 eV (C–O, C–N), 287.8 eV (C=O) and 289.0 eV (O–C=O). Also, a N 1s peak at 399.9 eV is attributed to the pyridine-like N atoms, revealing that the N atoms are successfully attached to the aromatic ring of the S-GQDs (Fig. 3d). These results disclose that an aromatic sp^2^ carbon network is formed, which is consistent with the result achieved with the FTIR spectrum.

**Fig. 4:**
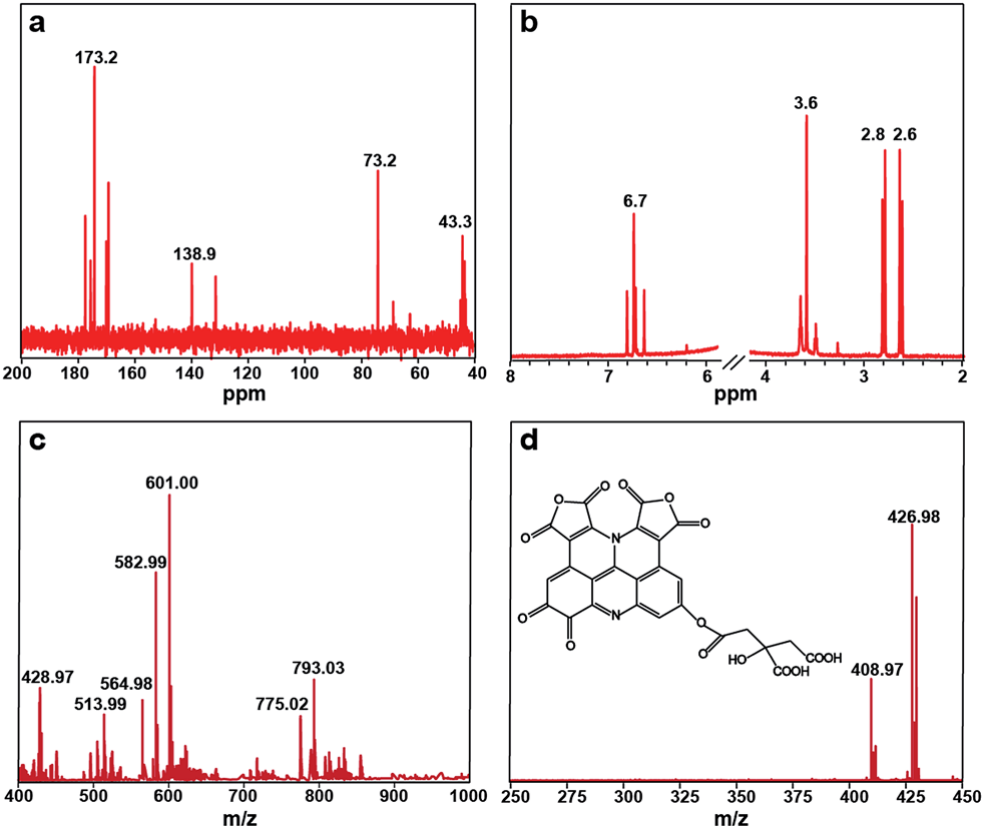
(a) ^13^C NMR spectrum and (b) ^1^H NMR spectrum of the S-GQDs; (c) negative-ion ESI-FTICR-MS of the S-GQDs; (d) MS/MS spectrum of *m*/*z* = 601.00. Inset is the structure of the S-GQDs corresponding to *m*/*z* = 601.00.

The ^13^C NMR spectrum of the S-GQDs in D_2_O solvent offers additional proof for the formation of a sp^2^ graphitic carbon network in the S-GQDs. As shown in Fig. 4a, the peaks from 40 to 80 ppm suggest that sp^3^ carbons are maintained, while the peaks from 100 to 180 ppm are attributed to sp^2^ carbons. Among those peaks assigned to sp^2^ carbons, the peaks between 120 and 130 ppm are most likely to be derived from (polycyclic) aromatic carbons,^16^ the peak at 138 ppm corresponds to C=C carbons, and the peaks between 160 and 180 ppm are ascribed to the carboxylic carbons.^17^ On the other hand, the ^1^H NMR spectrum of the S-GQDs is presented in Fig. 4b. The aromatic protons are found to be located at 6.5–7.0 ppm.^17*b*^ In a word, an aromatic carbon-rich architecture is formed in the S-GQDs.

The gel permeation chromatography (GPC) chromatogram of the S-GQDs equipped with a refractive index detector (Fig. S8†) displays a single peak with a retention time of 16.78 min, and the molecular weight is calibrated to be *M*_w_ = 589 based on a five point PEG standard calibration curve. Impressively, the narrow peak indicates a narrow size distribution of the S-GQDs, which relates well with the corresponding TEM results. In order to more accurately determine the molecular weight and the elementary composition of the S-GQDs, negative-ion ESI-FTICR-MS is used to characterize the S-GQDs in view of the existence of the carboxyl groups at their edges. Note that only one peak is observed at 589 Da in the GPC chromatogram, and thus the existence of higher and lower mass species is ruled out. We observed the main ions appearing at *m*/*z* = 601.00, 582.99, 564.98 (Fig. 4c), in which the ions *m*/*z* = 582.99 and *m*/*z* = 564.98 are obtained from the *m*/*z* = 601.00 after losing a molecule of H_2_O (18.01) and two molecules of H_2_O (36.02), respectively. To obtain exact structure information for the S-GQDs, tandem mass spectrometry (MS/MS) is used for the fragment *m*/*z* = 601.00 (Fig. 4d). Remarkably, a spacing unit of 174 Da is lost, reflecting the presence of the structure originated from a citric acid molecule (C_6_H_8_O_7_, 192 Da) after losing a molecule of H_2_O. These results clearly demonstrate that the S-GQDs contain the incompletely carbonized citric acid molecules after dehydrolysis under the hydrothermal conditions.^18^ In addition, the gradual loss of H_2_O molecules (18.01) from the MS/MS fragments at *m*/*z* = 601.00 implies that the structure of the S-GQDs contains hydroxyl groups. By virtue of the high-resolution elemental composition data, the ESI-FTICR-MS/MS fragmentation data, and the aforementioned spectral results, one possible structure mode of the native molecule with *m*/*z* = 601.00 is identified as C_27_H_10_N_2_O_15_, and its structure is shown in the inset of Fig. 4d. It is noteworthy that the edge structure of the S-GQDs is flexible although its conjugate planar is immobile. It should be also pointed out that the carbonization process is apparently complex and we do not intend to interpret the detailed fragmentation and the recombination process.

### Structure-directed photoluminescence properties of GQDs

Fig. 4d illustrates that the macrocyclic aromatic conjugation system constitutes the main units of structure of the S-GQDs. In general, the rigid planar cyclic structures possess blue photoluminescence properties.^19^ Interestingly, the as-prepared S-GQDs in this work exhibit a strong blue emission peak at 425 nm. It is concluded that the strong photoluminescence of the S-GQDs originates from the π–π electron transition of the rigid π-conjugate plane owing to the similar chemical structure of polyaromatic compounds.^20^ Therefore, the structure clarification of the S-GQDs provides direct evidence for the photoluminescence properties of the S-GQDs.

**Fig. 5:**
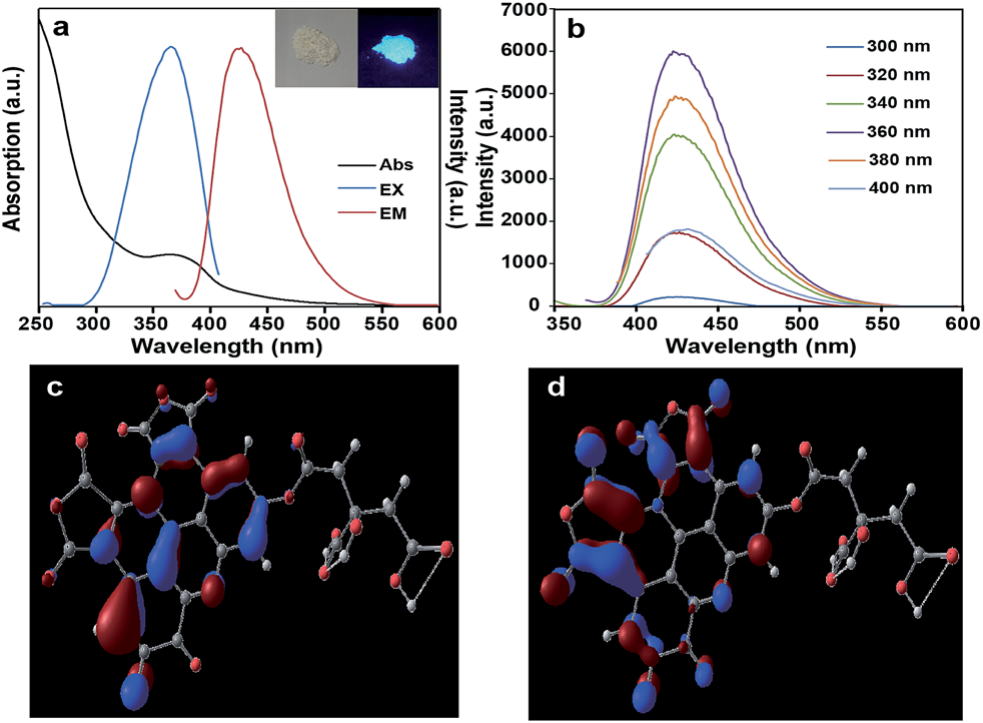
(a) UV-vis absorption spectrum and photoluminescence spectra of the Mg–Al–GQD-LDHs, inset are photographs of the Mg–Al–GQD-LDHs under visible (left) and UV light (right); (b) photoluminescence emission spectra of the Mg–Al–GQD-LDHs at different excitation wavelengths; (c) calculated HOMO (–6.668 eV) electron density distribution; (d) calculated LUMO (–4.284 eV) electron density distribution.

In the UV-Vis adsorption spectrum of the as-prepared Mg–Al–GQD-LDH solid composites (Fig. 5a), there is an absorption shoulder appearing at 365 nm. The inserted picture of the Mg–Al–GQD-LDHs sample displays bright blue light under UV light with excitation at 365 nm, corresponding to its emission spectra (Fig. 5b). The mirror image character between the excited and emission spectra is an indication of the rigid conjugated planar molecular structure of the as-prepared S-GQDs. The photoluminescence peaks of the solid Mg–Al–GQD-LDHs remain at 425 nm regardless of varied excitation wavelengths (Fig. 5b), and such an excitation-independent emission property highlights a high uniformity of the as-synthesized GQDs in size.^21^ Notably, the quantum yield of the as-prepared Mg–Al–GQD-LDHs is up to 40% under excitation at 365 nm, and the lifetime of the photoluminescence peak at 425 nm is calculated to be 10.77 ns (Fig. S9†).

Finally, the stability of the as-prepared GQD-LDHs is studied in a high salt and a wide pH range. It is interesting to observe that the equilibrium pH of the obtained Mg–Al–GQD-LDH colloidal solution remains almost constant (pH 8.0) upon increasing the initial pH from 4.0 to 10.0 (Fig. S10†). The good stability of the Mg–Al–GQD-LDHs in a wide pH range is ascribed to the strong buffering capacity of the LDHs.^22^ In addition, the photoluminescence emission of the Mg–Al–GQD-LDHs is highly salt resistant (Fig. S11†). These results reveal that the Mg–Al–GQD-LDHs can ensure the stability of their photoluminescence signals in real applications.

### Simulation structure of S-GQD

According to the above analytical results, the proposed chemical structure of S-GQD is subjected to structural optimization and spectral simulation by quantum chemistry calculations with Gaussian 09 software. The geometric structure is displayed in Fig. S12,† and the bond lengths and angles are listed in Table S1,† from which one may see that the C1–C13, C17–C22, N31, N32, O23–O25, O28–O30, O33, O34 atoms consist of a planar system, which is closely related to its optical properties.

It is well known that the highest occupied molecular orbital (HOMO) and lowest unoccupied molecule orbital (LUMO) determine the chemical and optical properties of a compound. The HOMO and LUMO energies of the S-GQD (C_27_H_10_N_2_O_15_) is calculated by B3LYP/6-31G(d) and shown in Fig. 5c and d and Table S2,† respectively. The main contribution to the HOMO is from the highest occupied C_6_H_8_O_7_ part, and the LUMO is largely caused by the C_21_H_3_N_2_O_8_ part. Interestingly, the HOMO is located over the benzene ring and the N atom, while the LUMO is registered over the furan-2,5-dione ring. This calculation result suggests that charge transfer exists in the S-GQD, which provides a potential application in optics. The HOMO and LUMO energies are –6.668 and –4.284 eV, respectively, so the energy gap between the HOMO and LUMO should be 2.384 eV (520 nm), being less than the observed photoluminescence energy (425 nm). Currently, the detailed reason for the blue shift of the photoluminescence peak is not clear, which might be caused by a calculation error, structure distortion or other factors affecting the HOMO–LUMO transition.

Spectral simulation is further carried out based on the optimized structure by the Time Dependent Density Functional Theory (TDDFT) method, and the calculated UV-Vis visible absorption (Fig. S13a†) and photoluminescence spectra (Fig. S13b†) show that this simulated structure has an optical absorption peak at 329 nm with two shoulders at 290 and 395 nm, and a photoluminescence peak at 381 nm with two shoulders at 347 and 425 nm. These results are qualitatively consistent with the observed UV-Vis absorption (365 nm) and photoluminescence spectra (425 nm) (please see Fig. 5a), which verifies that the proposed structure model of the S-GQD is correct.

## Conclusions

In summary, S-GQDs with well-defined structures have been controllably prepared by using hydrothermal pyrolysis of intercalated citrate within the 2D confined space of LDHs. A combination of both experimental study and theoretical simulation has been used to elucidate the intrinsic relationship between the structure and photoluminescence characteristics of the GQDs. Our success will broaden structure–function studies and speed up the deciphering of the photoluminescence code of GQDs. Efforts are underway to extend the generality of the confined space to govern the formation of other GQDs or carbon-based fluorescent materials, which will eventually advance our knowledge and give us the opportunity to control the building processes.
