# Supplementary material for: Structure observation of graphene quantum dots by single-layered formation in layered confinement space
Source: Chem Sci. 2015 Jun 4;6(8):4846–50. doi: 10.1039/c5sc01416f (PMC5502401; doi:10.1039/c5sc01416f)
Supplement: Supplementary file 1 [file SC-006-C5SC01416F-s001.pdf]

Electronic Supplementary Material (ESI) for Chemical Science.
This journal is © The Royal Society of Chemistry 2015

Electronic Supplementary Material (ESI) for Chemical Science
This journal is © The Royal Society of Chemistry 2014

Supporting Information for

Structure Observation of Graphene Quantum Dots by Single-Layered Formation in Layered Confinement Space

Liqing Song, Jingjing Shi, Jun Lu, and Chao Lu*

State Key Laboratory of Chemical Resource Engineering, Beijing University of Chemical Technology, Beijing 100029, China

Experimental section

Materials

All chemicals used were analytical-reagent grade and were used as received without any further purification. $Mg(NO_3)_2·6H_2O$, $Al(NO_3)_3·9H_2O$, NaOH, hydrochloric acid, citrate, ammonia, were purchased from Beijing Chemical Reagent Company (Beijing, China). Acetone was purchased from Thermo Fisher Scientific. The deionized water from a Millipore water purification system was used throughout the experiments.

Apparatus

The photoluminescence spectra were obtained using a F-7000 fluorescence spectrophotometer (Hitachi, Japan) at a slit of 2.5 nm with a scanning rate of 1500 nm/min. The solid UV-Vis absorption spectra were acquired on a Shimadzu UV-3600 spectrophotometer (Tokyo, Japan). Transmission electron microscopy (TEM) photographs were measured on a Tecnai G220 TEM (FEI Company, USA). High resolution transmission electron microscopy (HRTEM) was carried out on JEOL JEM-ARM200F at an acceleration voltage of 200 kV. X-ray photoelectron spectroscopy (XPS) measurements were performed using an ESCALAB-MKII 250 photo electron spectrometer (Thermo, USA). The lifetime was obtained by an Edinburgh FLS 980 Lifetime and Steady State Spectrometer. The quantum yield was measured with an integrating sphere from the reconvolution fit analysis (Edinburgh F980 analysis software) on an Edinburgh instrument spectrometer. ^{1}H nuclear magnetic resonance (^{1}H NMR) spectra and ^{13}C nuclear magnetic resonance (^{13}C NMR) were recorded at room temperature with a 600 MHz Bruker (Germany) spectrometer. The sample of S-GQDs was dissolved in D_2O. Electrospray ionization Fourier transform ion cyclotron resonance mass spectrometry (ESI-FTICR-MS) was carried out with solarix 9.4T (Bruker, Germany). Bruker Data Analysis was used to determine resolving power. The powder X-ray diffraction (XRD) measurements were performed on a Bruck (Germany) D8 Advance X-ray diffractometer equipped with graphite-monochromatized Cu/Kα radiation (λ = 1.54178 Å). The 2θ angle of the diffractometer was

This journal is © The Royal Society of Chemistry 2014
stepped from 5° to 70° at a scan rate of 10°/min. Fourier transform infrared (FTIR) measurements were performed using a Perkin Elmer Model 100 FTIR spectrometer (Waltham, MA, USA). Zeta potential measurement was determined using a Malvern Zetasizer 3000HS nano-granularity analyzer. Raman spectrum was gained by LabRAM ARAMIS Raman System (HORIBA Jobin Yvon, Japan) with 532 nm laser radiation source. Atomic force microscopy (AFM) in tapping mode was carried out on a NanoScope IIIa (Digital Instruments Co., Santa Barbara, CA, USA) instrument. Gel permeation chromatography (GPC) equipped with a Waters 1525 pump and a Waters 2414 refractive index detector (set at 35˚C).

Quantum chemistry calculation

The geometrical structure, the frontier molecular orbital analysis and the optical properties of the $C_{27}H_{10}N_2O_{15}$ were studied in detail. All calculations were performed using Gaussian 09 software package by parallel computing in the Linux system using the 16-cores server. First, the structure optimization was using B3LYP DFT method on 6-31G(d) basis set level, and the calculated frequency demonstrated that the optimized geometrical structure was stable. Based on the optimized geometries, the HOMO/LUMO analyses and spectral properties could be carried out. The UV-Vis absorption and photoluminescence spectra were computed by TDDFT.

Procedures

Synthesis of Mg-Al-citrate-LDHs

Mg-Al-citrate-LDHs were synthesized by a co-precipitation method in the solutions of constant pH value. A mixed salt solution containing 0.045 mol $Mg(NO_3)_2\cdot 6H_2O$ and 0.015 mol $Al(NO_3)_3\cdot 9H_2O$ in 60 mL water was added dropwise to a 60 mL solution containing 0.005 mol citrate at 30˚C under vigorous stirring. Subsequently, 2.0 M NaOH solution was added to keep a constant pH value of 10.5. Then, the suspensions were kept at 35˚C for 6 h under N_2 atmosphere. Finally, the precipitate was filtered, washed with deionized water for three times and stored at 4˚C until further use.

Synthesis of Mg-Al-GQD-LDHs

This journal is © The Royal Society of Chemistry 2014

Mg-Al-GQD-LDHs were synthesized by a hydrothermal method. 12.25 mL Mg-Al-citrate-LDHs (0.045 g/mL) and 2.25 mL ammonia were added into a Teflon-equipped stainless steel autoclave followed by hydrothermal treatment at 180˚C for 8 h. The resulting precipitate was separated by centrifugation (10000 rpm/min, 5 min), washed thoroughly with deionized water and dried in vacuum at 60˚C for 12 h. 2.25 g Mg-Al-GQD-LDHs were treated with 5 mL hydrochloric acid in order to dissolve the layers of LDH and obtain the pure solution of the S-GQDs. In addition, in order to remove inorganic salt, acetone was used to mix with the solution of S-GQDs. The precipitate was removed by centrifugation (10000 rpm/min, 5 min), and acetone in the supernatant was evaporated off under a stream of N_2, leaving a yellow brown powder of the S-GQDs.

This journal is © The Royal Society of Chemistry 2014

Table S1 Geometric optimized bond length and angle of the S-GQD ($C_{27}H_{10}N_2O_{15}$).

**Bond length（Å）**		**Bond angle (°)**	
C27-O28	1.381	O29-C27-C18	129.6
C18-C27	1.496	C27-C18-N31	127.1
C18-N31	1.377	C18-N31-C7	116.5
C7-N31	1.422	N31-C7-C3	121.0
C3-C7	1.395	C7-C3-C4	117.0
C3-C4	1.430	C3-C4-N32	124.5
C4-N32	1.316	N32-C4-C5	116.9
C5-O34	1.430	C4-C5-O34	121.8
C2-C17	1.209	O34-C5-C6	120.4
C17-C18	1.358	C1-C2-C17	124.6
C26-O30	1.196	C2-C17-C18	123.6
		C17-C26-O30	130.5
		O30-C26-O28	122.0
		C26-O28-C27	109.6

This journal is © The Royal Society of Chemistry 2014

Table S2 Frontier molecular orbital (HOMO and LUMO) of the S-GQD ($C_{27}H_{10}N_2O_{15}$).

**Compound**	$\mathbf{C_{27}H_{10}N_2O_{15}}$
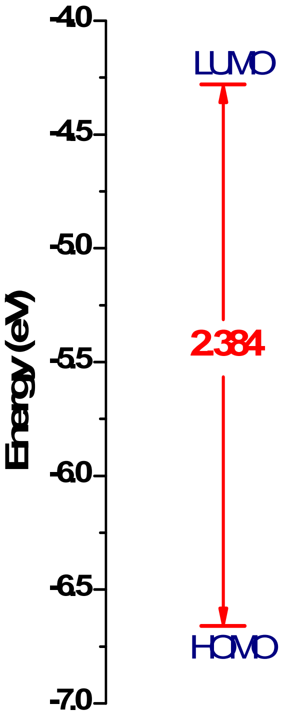	**LUMO(-4.284 eV)**
	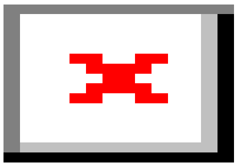
	**HOMO(-6.668 eV)**
	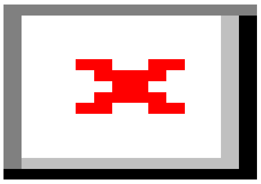

This journal is © The Royal Society of Chemistry 2014

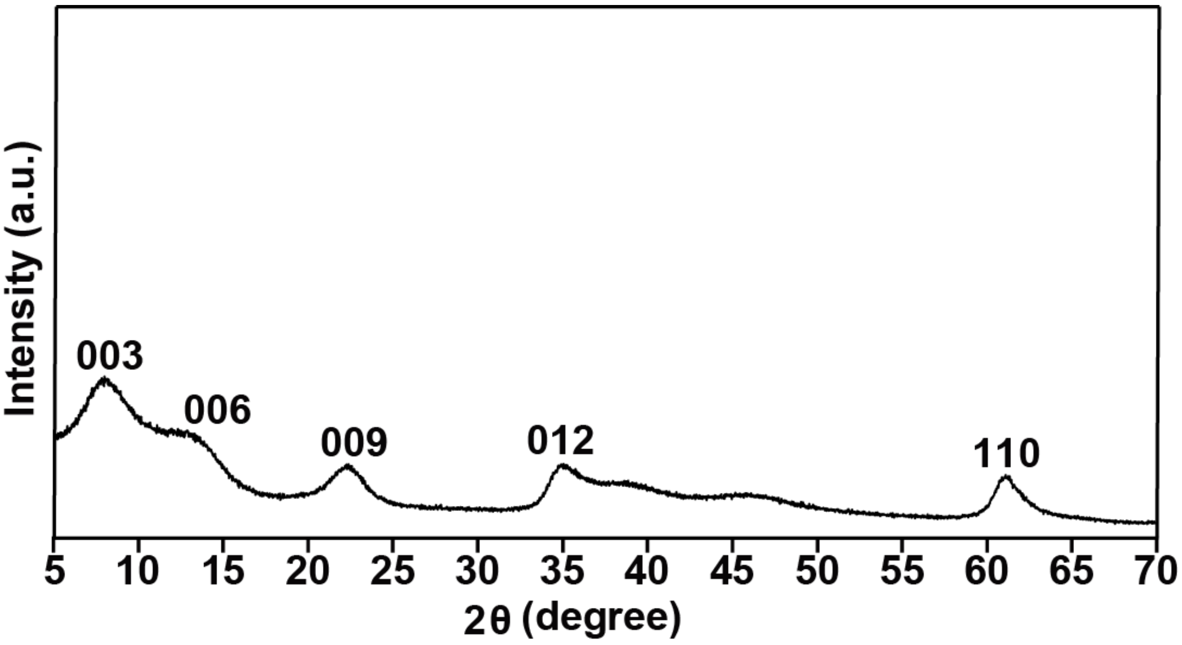

Figure S1. XRD pattern of Mg-Al-citrate-LDHs.

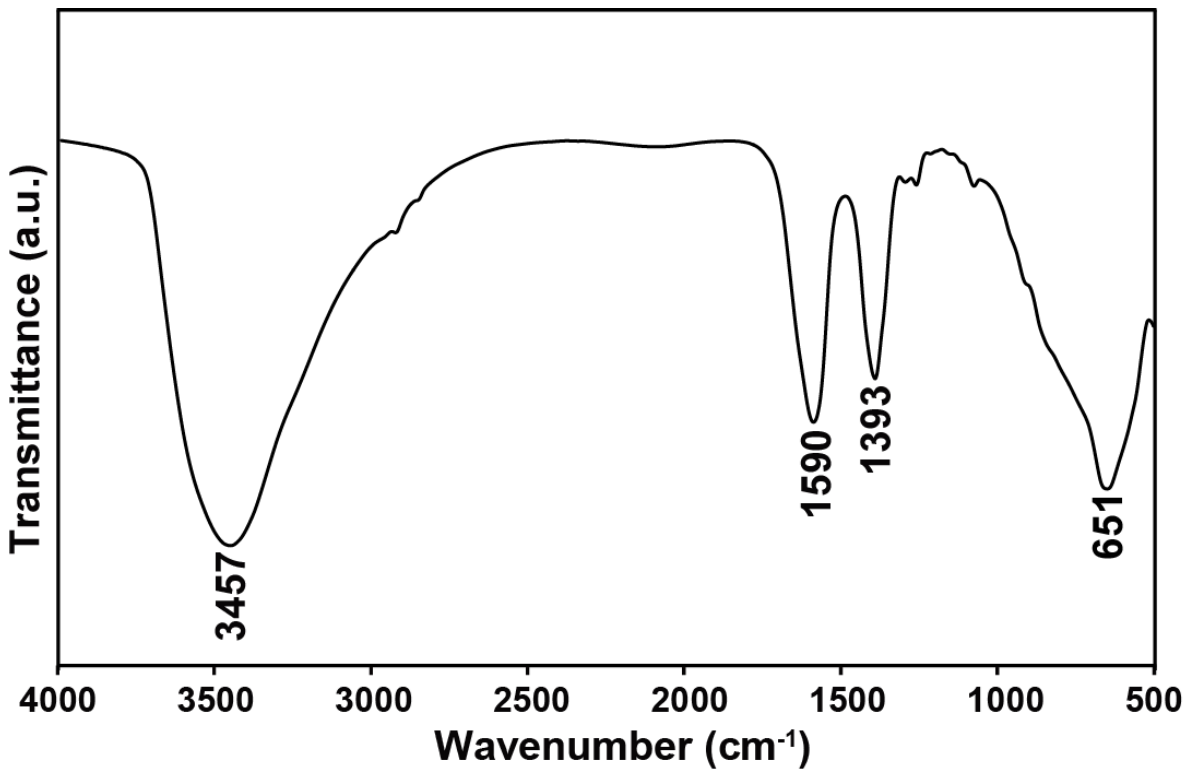

Figure S2. FTIR spectrum of Mg-Al-citrate-LDHs.

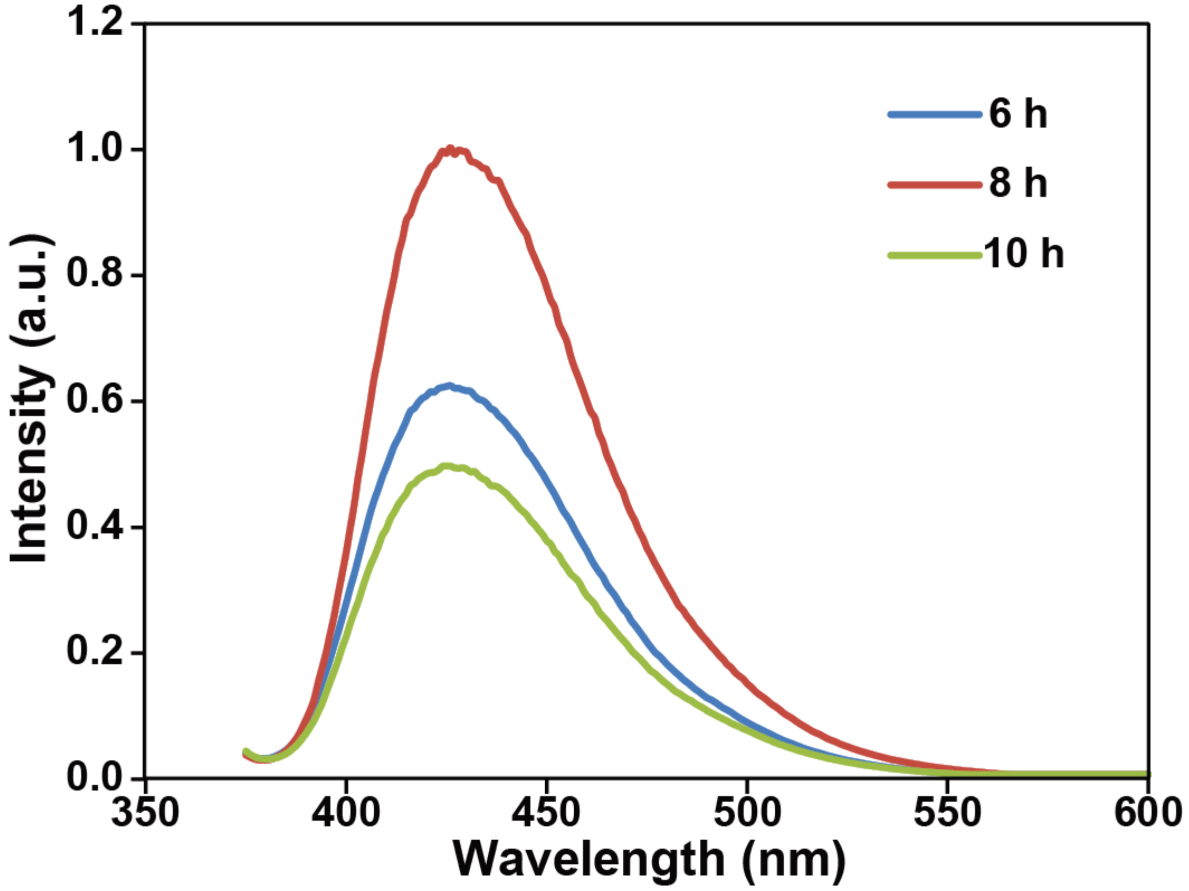

Figure S3. Photoluminescence emission spectra of Mg-Al-GQD-LDHs synthesized at different reaction time.

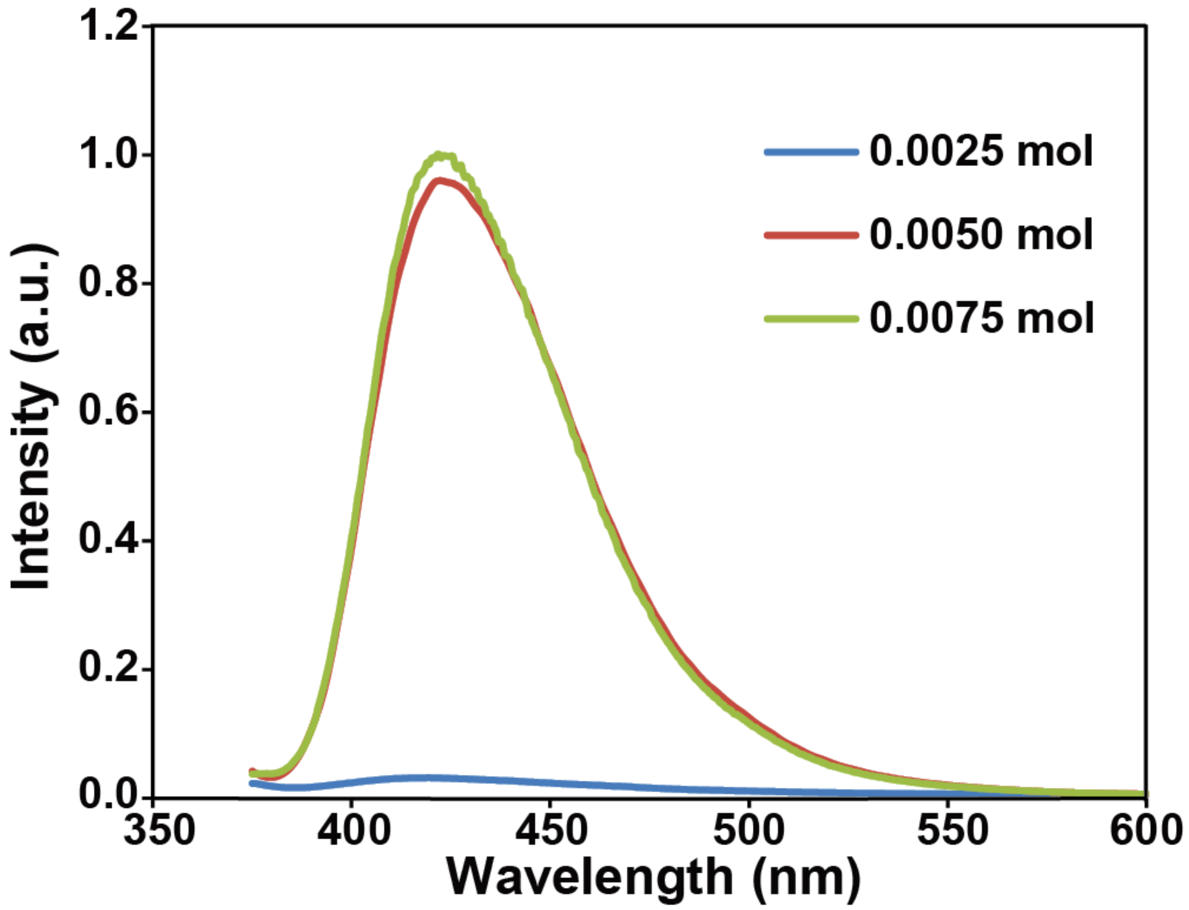

Figure S4. Photoluminescence spectra of Mg-Al-GQD-LDHs synthesized at 180˚C with different intercalated quantity of citrate.

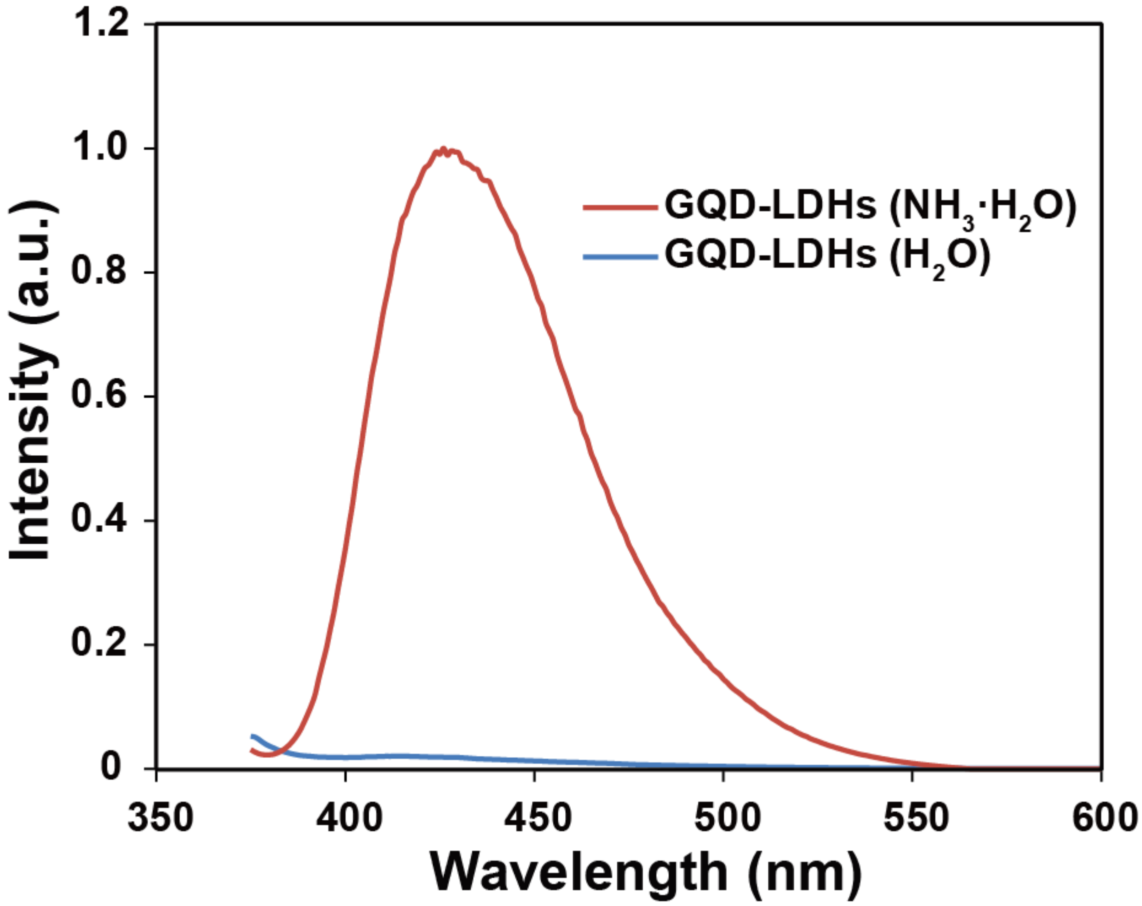

Figure S5. Photoluminescence emission spectra of Mg-Al-GQD-LDHs synthesized with and without ammonia.

This journal is © The Royal Society of Chemistry 2014

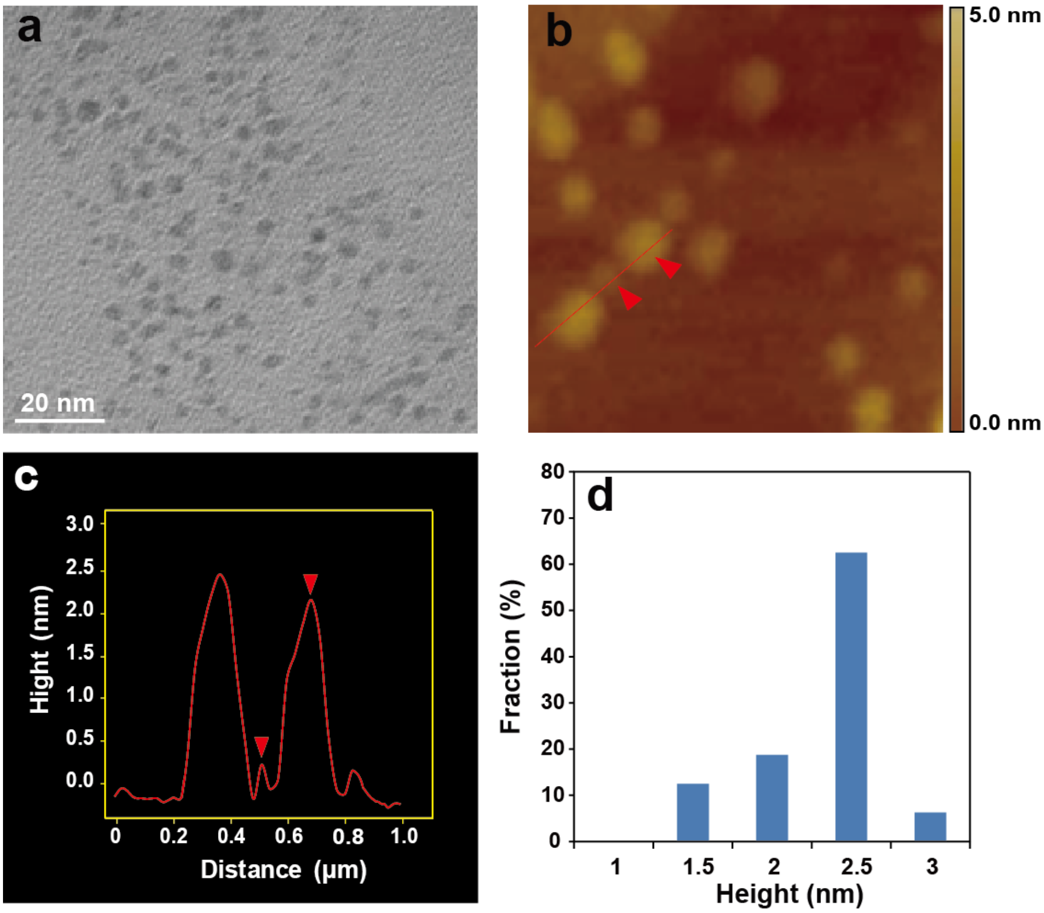

Figure S6. (a) TEM image of the GQDs prepared from pure citrate; (b) AFM image of GQDs; (c) Height profile along the red line in (b); (d) Height distributions of GQDs.

This journal is © The Royal Society of Chemistry 2014

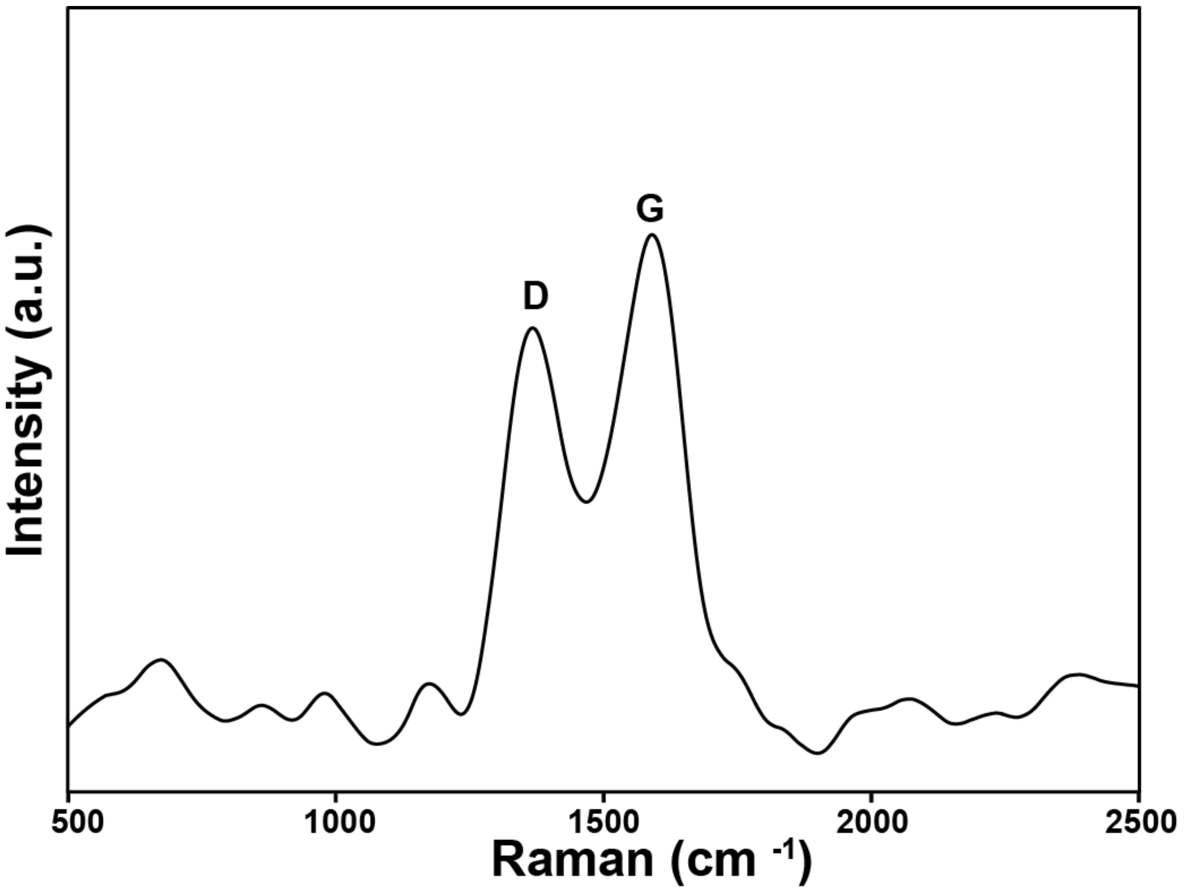

Figure S7. Raman spectrum of GQDs.

This journal is © The Royal Society of Chemistry 2014

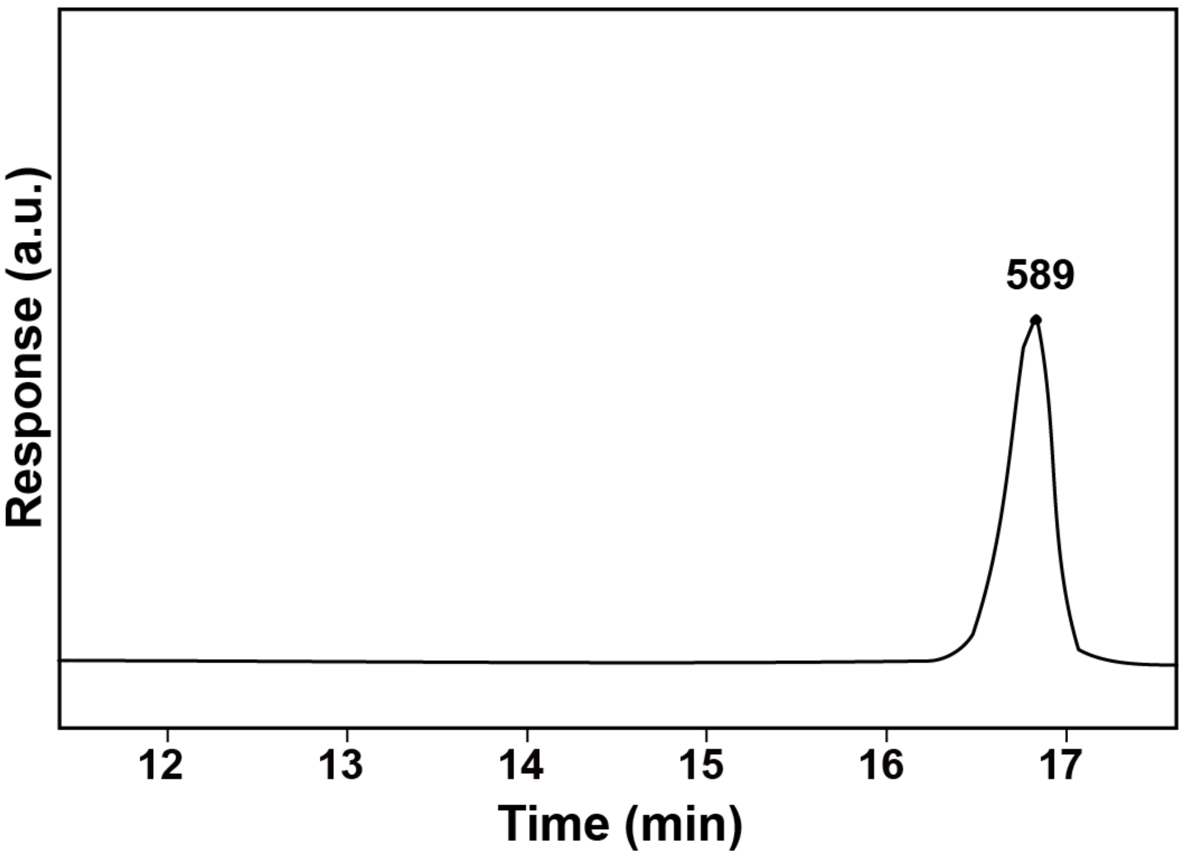

Figure S8. GPC chromatogram of S-GQDs.

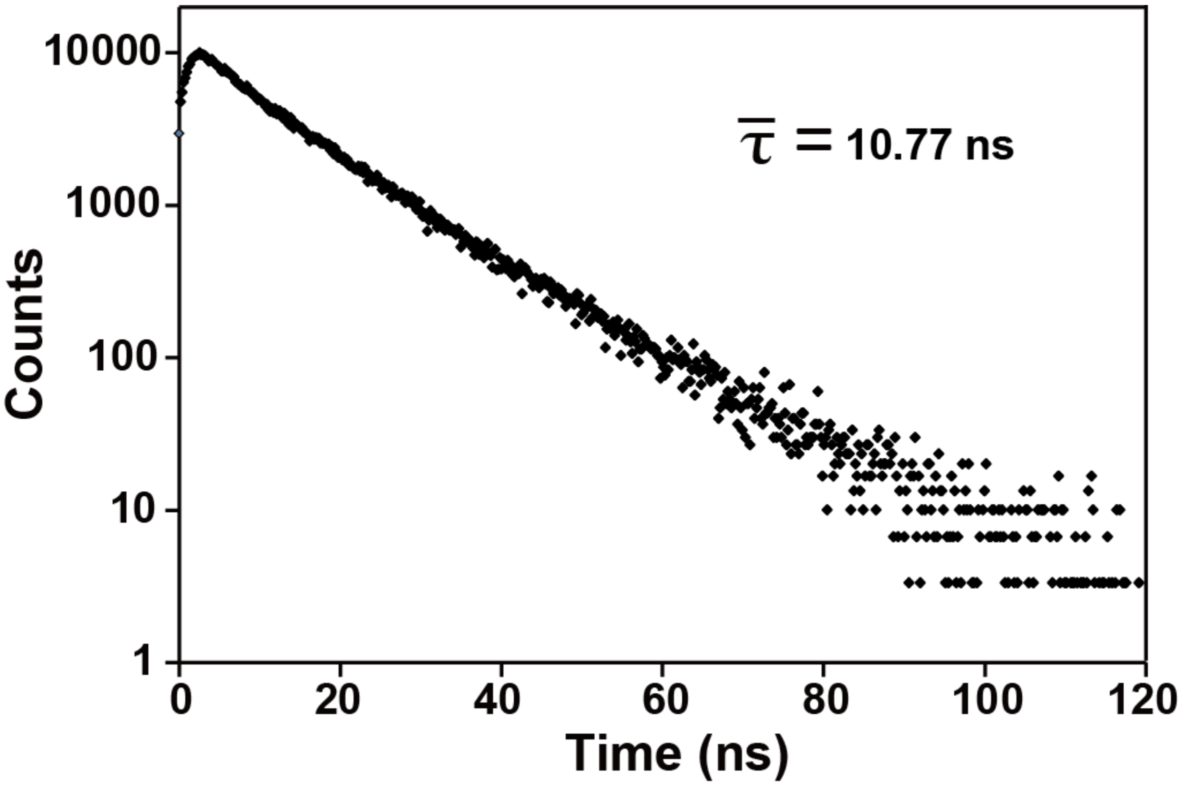

Figure S9. The time-resolved photoluminescence curve of the Mg-Al-GQD-LDHs.

This journal is © The Royal Society of Chemistry 2014

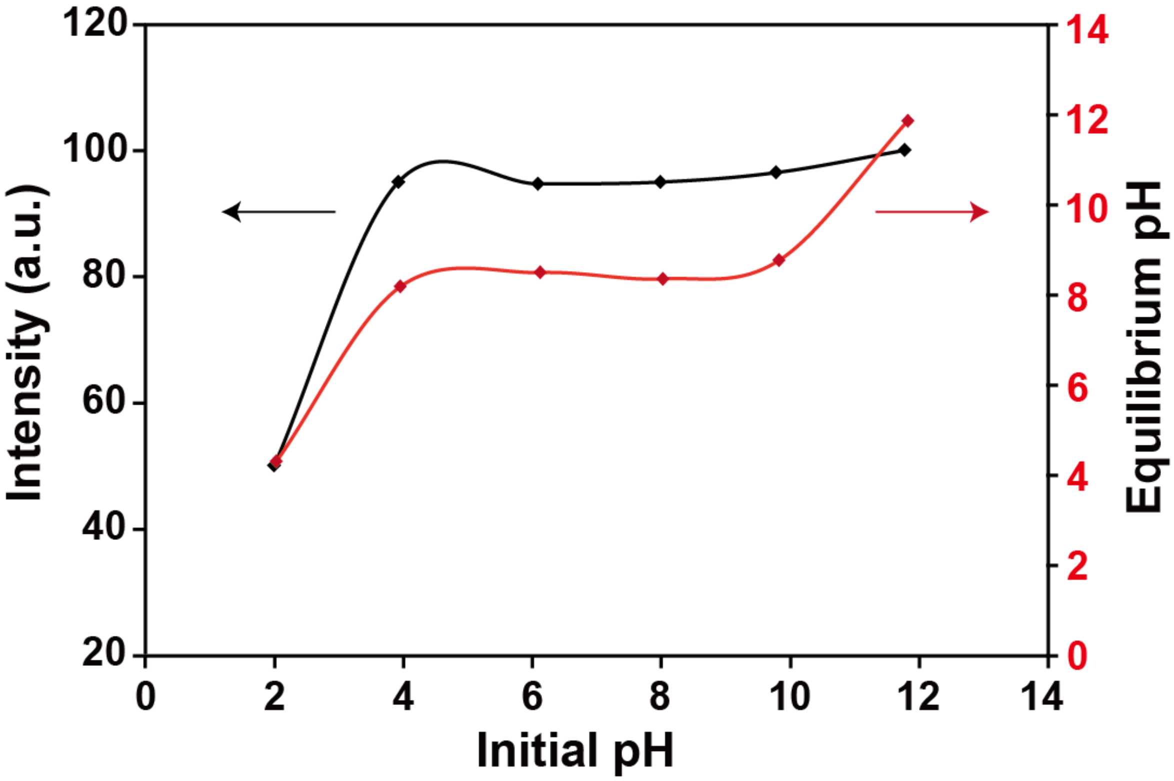

Figure S10. Effect of pH on the photoluminescence intensity of Mg-Al-GQD-LDHs. Mg-Al-GQD-LDHs were added into the different pH solutions with continuous stirring for 30 min and the equilibrium pH of the solution was measured. (black points: photoluminescence intensity versus initial pH values; red points: equilibrium pH values versus initial pH values).

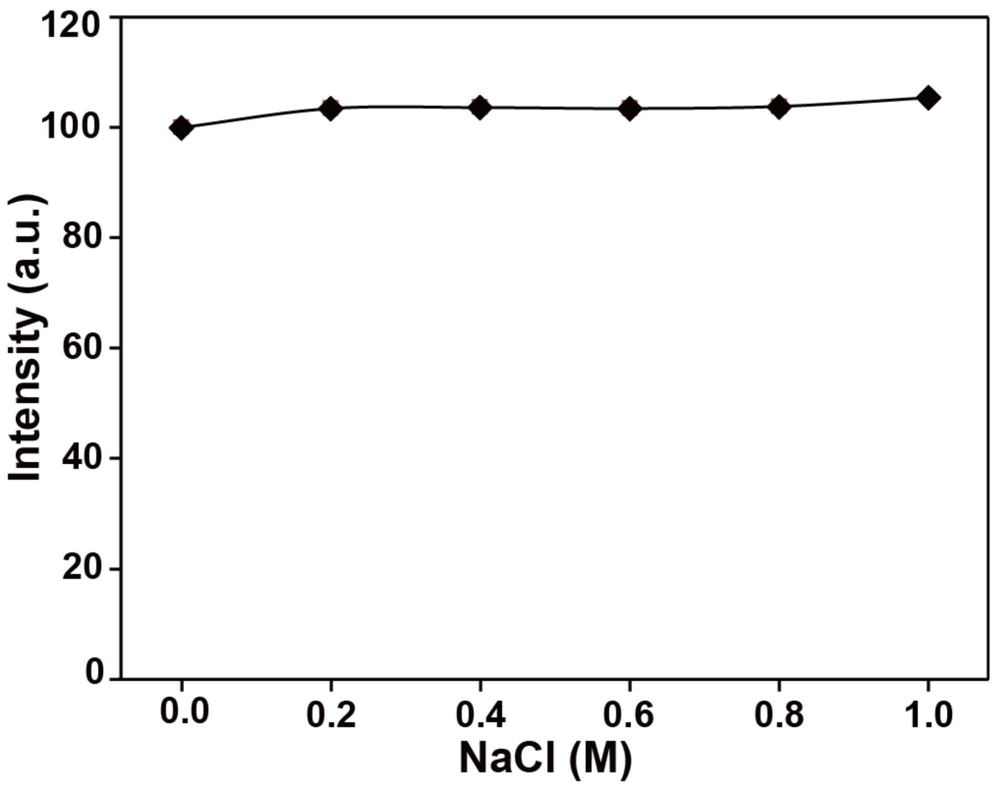

Figure S11. Effect of salt concentration on the photoluminescence intensity of Mg-Al-GQD-LDHs (λ_{ex} = 365 nm).

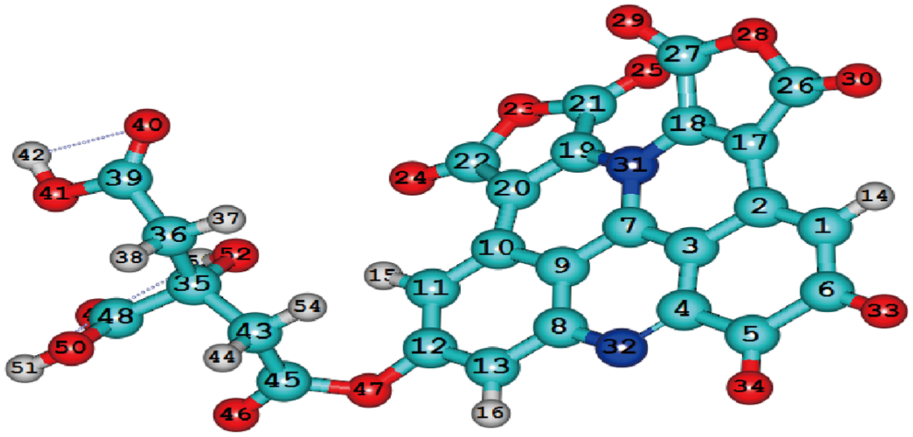

Figure S12. The geometric optimized structure of the S-GQD by Gaussian 09 calculation. The cyan, red, blue and grey solid balls represent the carbon, oxygen, nitrogen and hydrogen atoms, respectively.

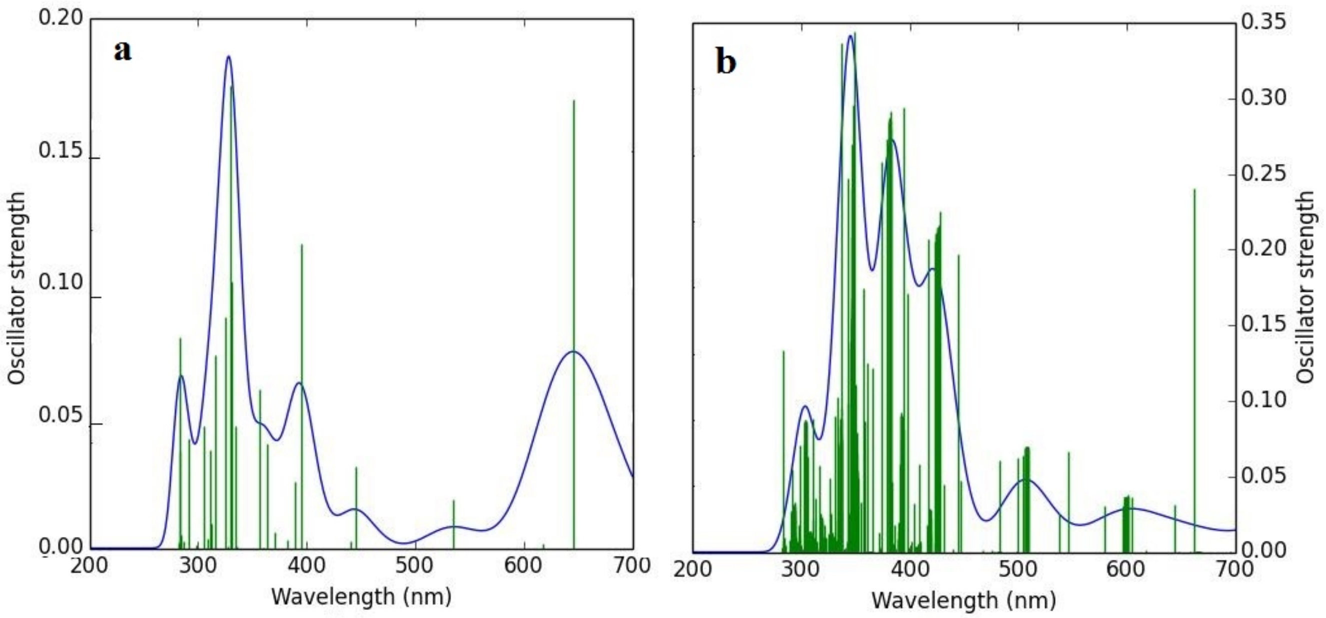

Figure S13. The optical absorption spectrum (a) and photoluminescence spectrum (b) of the S-GQD calculated by the TDDFT method. Note that the optimized structure of S-GQD is based on the B3LYP/6-31G(d) method.
